# Effects of Angiotensin II Type 1 receptor blocker and adiponectin on adipocyte dysfunction in stroke-prone spontaneously hypertensive rats

**DOI:** 10.1186/1476-511X-12-108

**Published:** 2013-07-22

**Authors:** Kumiko Takemori, Takao Inoue, Hiroyuki Ito

**Affiliations:** 1 Department of Food Science and Nutrition, Faculty of Agriculture, Kinki University 3327-204, Nakamachi Nara-City, Nara 631-8505, Japan; 2 Department of Pathology, Faculty of Medicine, Kinki University 377-2 Ohno-Higashi, Osaka-Sayama, Osaka 589-8511, Japan; 3 Department of Biomedical Engineering, Faculty of Biology-Oriented Science and Technology, Kinki University 930 Nishi-mitani, Kinokawa, Wakayama 649-6493, Japan

**Keywords:** Stroke-prone spontaneously hypertensive rats, Adipose tissue, Renin–angiotensin system, Angiotensin II type I receptor blocker, Lipoatrophy, Adipokines

## Abstract

**Background:**

Hypoadiponectinemia in lipoatrophy may be related to worsening of hypertension in stroke-prone spontaneously hypertensive rats (SHRSP). One of the beneficial effects of candesartan (Angiotensin II Type 1 receptor blocker) for preventing hypertension may be increasing of adiponectin due to improvement of adipocyte dysfunction. In this study, we determined the effects of candesartan or adiponectin on pathophysiologic features and adipocyte dysfunction in SHRSP.

**Methods:**

Candesartan was administered to male SHRSP from 16 to 20 weeks of age (2 mg/kg/day). Adiponectin was cloned and intravenously administered to male SHRSP from 16 to 20 weeks of age. We examined biological parameters, as well as the expression and release of adipokines.

**Results:**

The SHRSP exhibited severe atrophy of visceral fat and progression of severe hypertension. The expression and release of leptin and adiponectin were impaired at 6 and 20 weeks of age. Candesartan suppressed the development of lipoatrophy and reduced the incidence of stroke at 20 weeks of age. Candesartan also enhanced the expression of adiponectin and leptin by inducing the overexpression of peroxisome proliferator activated receptor γ. Circulating level of leptin was significantly higher in candesartan group than in the control group, whereas adiponectin was similar in both groups. Intravenous administration of adiponectin resulted in enhancement of adiponectin expression in adipose tissue, but no remarkable effects were found in pathophysiology in SHRSP.

**Conclusions:**

Our results indicate that candesartan protects against hypertension and adipocyte dysfunction in SHRSP. The induction of leptin expression appeared to be important factor in the inhibition of stroke lesions, whereas adiponectin was not a major regulator of blood pressure in SHRSP with genetic hypertension. Further studies are needed to elucidate the role of the renin–angiotensin system in adipose tissue dysfunction in relation to hypertensive end-organ damage.

## Background

As is well known, adipose tissue is a major endocrine system, and the abnormal expression of adipokines by hypertrophied adipocytes in obesity is involved in the pathogenesis of hypertension and vascular disorders [1]. On the other hand, an association between hypertension and lipoatrophy was also reported in patients infected with human immunodeficiency virus [2,3] and in patients with familiar partial lipodystrophy (FPLD3) [3,4].

Stroke-prone spontaneously hypertensive rats (SHRSP), a well-established model of genetic hypertension, are lean and develop marked lipoatrophy with the progress of hypertension. Although several factors are involved in the pathogenesis of severe hypertension in SHRSP, lipoatrophy may be an important factor, because feeding these rats a high-fat high-cholesterol diet improved lipoatrophy, extended their life-span, and decreased the incidence of stroke (unpublished data). These results suggest that adipocyte dysfunction is related to hypertension in SHRSP. Moreover, A-ZIP/F-1 mice, an animal model of lipoatrophic diabetes, lack white adipose tissue and exhibit hypertension [5]. We previously reported that A-ZIP/F1 mice show higher blood pressure and enhanced agonist-induced arterial wall contractility compared with wild-type mice [6]. These findings suggest that adipose tissue may have an important role in the pathogenesis of hypertension in obesity and in lipoatrophy.

Since Engeli et al. [7] first reported that the renin-angiotensin system (RAS) is localized in many tissues, including adipose tissue, many studies have been performed to examine the role of the RAS in adipose tissue in relation to adipocyte differentiation and hypertension. For example, Zorad et al. [8] investigated the effects of candesartan, an angiotensin II type I receptor blocker (ARB), on adipose tissue growth, and adipokine expression and release in normotensive non-obese Wistar–Kyoto rats (WKY). They reported that candesartan induced adipose tissue hypotrophy and increased the expression of adiponectin and peroxisome proliferator-activated receptor (PPAR)γ. We previously reported that early and transient treatment of SHRSP with low doses of candesartan prevented hypertensive end-organ damage [9,10]. The protective effects of ARBs against hypertension and its complications were clearly demonstrated in a comprehensive series of studies performed by Saavedra et al. [11-13]. Their and our findings suggest that these beneficial effects of ARB may be due to prolonged inhibition of the RAS in several tissues, including adipose tissue. However, the mechanisms involved in these effects of RAS inhibition were unclear.

In this study, we focused on the pathophysiological characteristics of visceral adipose tissue in SHRSP. As one of the beneficial effects of candesartan, we hypothesized that candesartan may improve circulatory disturbances in SHRSP by enhancing adiponectin production as a result of inhibiting the RAS in adipose tissue. To test this hypothesis, we treated SHRSP with either candesartan or adiponectin and determined the changes in blood pressure and adipocyte function.

## Results

### Comparison of adipocyte function between WKY and SHRSP

We first assessed the adipokine expression profiles in WKY and SHRSP at 6 and 20 weeks of age. Table 1 shows the serum adipokine levels and mRNA expression in epididymal adipose tissue. At 6 weeks of age, the serum leptin and adiponectin levels were significantly lower in SHRSP than in WKY. Furthermore, the mRNA expression levels of leptin, adiponectin, and PPARγ were lower in SHRSP, consistent with the circulating levels. The circulating leptin and adiponectin levels were also lower in SHRSP than in WKY at 20 weeks of age. Although the mRNA expression level of leptin in epididymal fat tissue was significantly lower in SHRSP than in WKY, there were no significant differences in either adiponectin or PPARγ mRNA expression levels between WKY and SHRSP. These results indicate that adipocyte dysfunction occurred before the onset of hypertension in SHRSP. Based on these findings, we conducted experiments to examine the effects of treating SHRSP with an ARB.

**Table 1:** **Circulating and mRNA expression levels of adipocyte**-**related molecules in WKY and SHRSP**

	**6 weeks of age**		**20 weeks of age**	
	**WKY**	**SHRSP**	**WKY**	**SHRSP**
**Serum adipokine levels**				
Leptin (ng/ml)	2.895±0.700	0.568±0.450	9.003±0.673	4.497±0.383**
Adiponectin (μg/ml)	11.559±1.037	8.491±0.243**	10.054±0.219	5.934±0.222**
**Adipokine expression in epididymal adipocytes (ratio to 18S rRNA)**				
Leptin	1.059±0.098	0.251±0.049**	4.204±0.746	2.012±0.177**
Adiponectin	1.045±0.070	0.609±0.050*	1.301±0.108	1.167±0.05
PPARγ	1.010±0.086	0.544±0.068*	0.517±0.038	0.407±0.050

SHRSP: stroke-prone spontaneously hypertensive rats; PPARγ: peroxisome proliferator-activated receptor γ; WKY: Wistar–Kyoto rats. Values are means ± SE. *p < 0.05 and **p < 0.01 vs. age matched WKY (Student’s *t*-test).

### Effects of candesartan on blood pressure, body weight, and cerebral lesions

Table 2 presents the biological characteristics of 20-week-old SHRSP treated with or without candesartan. Blood pressure was significantly lower and body weight was significantly heavier in the candesartan-treated group than in the control group. The total visceral fat pad weight was significantly greater in the candesartan-treated group than in the control group. By contrast, brain weight was significantly lower in the candesartan-treated group than in the control group. Cerebral lesions (edema, softening, or hemorrhage) were found in 7/8 rats (87.5%) in the control group but in none in the candesartan-treated group.

**Table 2:** Effects of candesartan on biological parameters in SHRSP

	**Control group**	**Candesartan group**	**p**
Blood pressure (mmhg)	265±5	213±6	<0.01*
Body weight (g)	287±8	307±6	<0.05*
Brain weight (g)	2.061±0.052	1.897±0.023	<0.05*
Stroke (rats with stroke/total rats)	7/8 (87.5%)	0/8 (0%)	<0.01#
Total fat pad (g)	6.242±0.563	9.344±0.338	<0.05*

Values are means ± SE. *Student’s *t*-test; #Wilcoxon’s test.

### Effects of candesartan on adipokine expression

**Figure 1:**
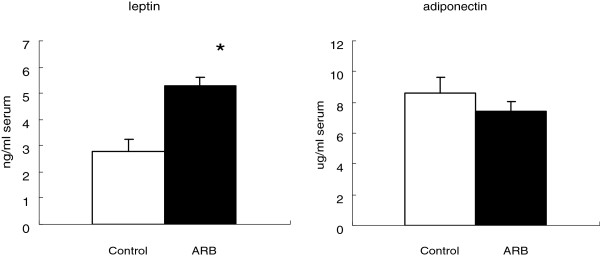
**Effects of candesartan on plasma leptin and serum adiponectin levels.** Values are means ± SE. *p < 0.05 vs. the control group.

As shown in Figure 1, plasma leptin levels were significantly higher in the candesartan group than in the control group, whereas serum adiponectin levels were similar in both groups. However, the mRNA expression levels of leptin, adiponectin, and PPARγ in adipocytes were significantly greater in the candesartan group than in the control group, as indicated in Table 3.

**Table 3:** **Effects of candesartan on mRNA expression levels of adipocyte**-**related molecules**

	**Control group**	**Candesartan group**	p*
Leptin	0.17±0.02	0.36±0.05	<0.01
Adiponectin	0.53±0.06	0.94±0.11	<0.01
PPARγ	0.11±0.02	0.34±0.07	<0.01

PPARγ: peroxisome proliferator-activated receptor γ. Expression levels are shown as the ratio to 18*S* rRNA. Values are means ± SE. *Student’s *t*-test.

### Effects of intravenous adiponectin administration

Table 4 shows the effects of continuous intravenous infusion of adiponectin in SHRSP. There were no significant differences in blood pressure or body weight between the adiponectin-treated group and the control group. Blood adiponectin levels were slightly, but not significantly, higher in the adiponectin-treated group than in the control group, whereas leptin levels were similar in both groups. The mRNA expression level of adiponectin in adipose tissue was significantly higher in the adiponectin-treated group than in the control group. However, the expression levels of adiponectin receptors (Adipo-R1, Adipo-R2, and cadherin 13) in skeletal muscle were not significantly different between the two groups.

**Table 4:** Effects of intravenous adiponectin infusion in SHRSP

		**Control group**	**Adiponectin group**	p*
Blood pressure	(mmHg)	241±3	249±7	ns
Body weight	(g)	305±7	303±8	ns
**Blood**				
Adiponectin	(ng/ml)	7160±680	7974±388	ns
Leptin	(pg/ml)	2593±807	2331±363	ns
**Adipose tissue**				
Adiponectin	Ratio to 18SrRNA	0.23±0.02	0.51±0.06	<0.01
**Muscle**				
Adipo-R1	Ratio to 18SrRNA	0.97±014	0.95±0.06	ns
Adipo-R2		0.99±0.13	0.76±0.09	ns
Cadherin 13		22.29±3.89	16.72±1.35	ns

Adipo-R: adiponectin receptor. Values are means ± SE. *Student’s *t*-test.

## Discussion

In the present study, we revealed that candesartan ameliorated not only hypertension but also lipoatrophy in SHRSP. One of the mechanisms of decreasing of blood pressure and lowering of stroke lesion may be due to improvement of adipocyte dysfunction.

Although angiotensin II was reported to inhibit adipocyte differentiation [14,15], angiotensin II-induced lipolysis was ameliorated by the ARB losartan [16]. The angiotensin II type 1 and type 2 receptors are involved in determining adipocyte size [8,17]. Thus, the RAS in adipose tissue is closely related to adipocyte growth and function. Accordingly, angiotensin II signaling via the type 1 and type 2 receptors may regulate adipocyte particle size, and ARBs may ameliorate adipocyte dysfunction.

In this study, plasma leptin levels were higher in the candesartan group than in the control group, but adiponectin levels were similar in both groups. However, the mRNA expression levels of leptin, adiponectin, and PPARγ were higher in the candesartan group than in the control group.

Leptin is secreted from hypertrophic adipocytes and its circulating level is directly proportional to body fat. In terms of its roles, leptin stimulates energy consumption by activating the sympathetic nerve system and promotes fatty acid β-oxidation in peripheral tissues [18]. Plasma leptin and epididymal leptin mRNA levels were higher in the candesartan group than in the control group. This increase in leptin expression possibly contributed to the lower incidence of stroke in the candesartan-treated group. Although precise mechanisms are still unknown, leptin may protects against severe energy depletion due to lopatrophy in SHRSP. This possibility is supported by a study conducted by Avraham et al. [19], who reported that leptin administration significantly improved neurological disability and reduced infarct volume following permanent middle cerebral artery occlusion in Sebra mice. Furthermore, in clinical trials, it was reported that leptin improved insulin resistance in lipoatrophic patients [20,21]. Although further large-scale trials are needed to demonstrate the long-term efficacy and safety of leptin, a new drug application for leptin is anticipated [22]. To explore the therapeutic role of leptin, Ebihara et al. [23] crossed A-ZIP/F1 mice, which exhibit severe lipoatrophy, with mice overexpressing leptin in the liver. The resulting doubly transgenic mice virtually lacked adipose tissue but had elevated leptin levels. These mice showed improvements in insulin sensitivity, hepatic steatosis, and normal glucose and insulin levels [23]. These results indicate that leptin has an important role in the pathogenesis of circulatory disturbance as well as metabolic disorders.

On the other hand, the mRNA expression levels of adiponectin and PPARγ were also increased in the candesartan-treated group. PPARγ is nuclear transcription factor in adipocytes, where it control the expression of numerous genes involved in glucose and lipid metabolism, and cell differentiation in particular [24]. Zorad et al. [8] investigated the effects of candesartan in normotensive WKY and reported marked decreases in body weight with increasing of expression of adiponectin and PPARγ. However, the plasma leptin level and its mRNA expression in adipose tissue were lower in the candesartan group than in the control group. Their findings were generally similar to those of our present study, except for leptin expression. This difference may be due to differences in methods, including strain of rat, candesartan dose, and the duration of treatment. Nevertheless, the results of both studies are consistent in terms of the improvement in adipocyte function in candesartan-treated rats.

As described above, candesartan ameliorated adipocyte dysfunction in SHRSP, which we thought were mediated by an increase in adiponectin production. To confirm this hypothesis, we investigated the effects of continuous intravenous infusion of recombinant adiponectin in mature SHRSP. The results of this experiment revealed an increase in plasma adiponectin levels, although the increase was not statistically significant. However, the adiponectin mRNA expression level in adipose tissue was significantly higher in the adiponectin-treated group than in the control group. On the other hand, there were no significant differences in the mRNA expression levels of leptin, Adipo-R1, AdipoR-2, or cadherin 13 between the two groups. Unexpectedly, we found no differences in body weight or blood pressure between the two groups. Bassi et al. [25] investigated the chronic effects of centrally administered adiponectin in SHR and reported that adiponectin did not appear to have an important role in long-term control of blood pressure. Thus, we and Bassi et al. [25] found no remarkable effects of adiponectin on the circulatory disturbances in SHR or SHRSP. This means that adiponectin is not a major regulator of blood pressure in essential (i.e., genetic) hypertension. However, several reports have indicated that adiponectin may lower blood pressure by regulating the nitric oxide synthetic pathway [26,27]. We did not investigate the changes in nitric oxide-related molecules in the present study.

In conclusion, we revealed the candesartan has beneficial effects not only for decreasing of blood pressure but also improvement of adipocyte dysfunction, although the latter must be secondary to decreasing of blood pressure. Leptin may be more deeply related to decreasing of stroke as compared to adiponectin in SHRSP. Further studies are required to elucidate t role of renin-angiotensin system in adipocyte in relation to hypertension and stroke.

## Materials and methods

### Animals

Male SHRSP/kpo and Wistar–Kyoto rats (WKY/kpo), as a normotensive control, were used in this study. These rats are from the original strains provided by Dr. Okamoto (Kyoto University) and were maintained at the Animal Center, Faculty of Medicine, Kinki University. All experimental protocols conformed to the guidelines of the National Institutes of Health (Guide for the Care and Use of Laboratory Animals 1996), and were approved by the Institutional Animal Experimentation Committee of Kinki University.

### Characterization of SHRSP and WKY

Male SHRSP and WKY were used at 6 and 20 weeks of age (6 rats in each). The biological factors (as described below), serum adipokine levels, and the mRNA expression levels of adipocyte-related genes in epididymal fat tissue were determined.

### Effects of candesartan administration

Male SHRSP at 15 weeks of age were randomly divided into a control group and a candesartan-treated group (eight rats per group). Candesartan (Takeda Pharmaceutical Co. Ltd., Tokyo, Japan) was orally administered for 5 weeks from 16 weeks of age (2 mg/kg/day).

### Effects of adiponectin administration

To investigate the effects of exogenous adiponectin on the pathophysiology in SHRSP, we first cloned rat adiponectin using *Escherichia coli*, as described below. Purified adiponectin (in phosphate-buffered saline [PBS]) was administered to five 16-week-old SHRSP for 2 weeks using an osmotic mini-pump (100 μg/day). Control rats (*n* = 5) were administered PBS alone (n = 5).

### Measurements

Blood pressure and body weight was measured once weekly until 20 weeks of age. Blood pressure was measured using the tail-cuff method in conscious rats. At 20 weeks of age, blood samples were taken from the vena cava under sodium pentobarbital anesthesia. Plasma samples were prepared from blood collected in heparinized syringes by centrifugation at 6,000 × *g* for 10 min. Serum samples were prepared from blood collected in non-heparinized syringes by centrifugation at 10,000 × *g* for 10 min. Brain and epididymal fat tissue were excised, weighed, and observed macroscopically.

### Molecular cloning, expression, and purification of full-length rat adiponectin

The adiponectin gene (*Adipoq*, NM_144744) was amplified from cDNA prepared using the reverse primer 5’-ACCCAAGCTTCAGTTGGTATCATGGTAG-3’. The resulting PCR product was digested with *Bam*HI and *Hin*dIII, and then cloned into a pQE30 vector (Qiagen, Hilden, Germany), which has 6× His tag at the N terminus the adiponectin gene. The construct was then transformed into *E*. *coli* JM109 (New England BioLabs, Ipswich, MA, USA) and screened on Luria broth medium containing 100 μg/ml ampicillin (LB-Amp). The plasmid was collected from LB-Amp–screened *E*. *coli* JM109 and the *Adipoq* sequence was screened using an ABI PRISM® 310 Genetic Analyzer (Applied BioSystems, Foster City, CA, USA).

*E*. *coli* JM109 containing the *Adipoq* gene were cultivated in LB-Amp at 37°C by inducing adiponectin protein expression with isopropyl-β-D-thiogalactopyranoside. The bacteria were harvested and stored at −20°C until use. The *E*. *coli* were then solubilized with lysis buffer containing BugBuster (Takara Bio, Shiga, Japan), 100 mM imidazole, and protease inhibitor cocktail (Sigma, St. Louis, MO, USA) in PBS (pH 7.4) at room temperature for 30 min, followed by centrifugation at 16,000 × *g* for 20 min. The precipitates were collected because adiponectin protein was stored in inclusion body (data not shown). The inclusion bodies were isolated using BugBuster, and then solubilized and denatured with 2 ml of PBS (pH 7.4) containing 7 M guanidine-HCl and 1% (v/v) α-mercaptoethanol at room temperature. The denatured adiponectin was refolded by incubation in a 200-fold volume of PBS containing 2 M urea at 4°C for 3 days. After concentration, the adiponectin was purified using an Ni Sepharose™ 6 Fast Flow column (GE Healthcare, Little Chalfont, UK) by eluting with PBS containing 200 mM imidazole. The purified adiponectin was cleared of endotoxin using ActiClean Etox (Sterogene Bioseparations Inc., Carlsbad, CA, USA), concentrated, transferred to PBS, and passed through a 0.22 μm filter.

### Analysis of adipokine levels

Plasma leptin and serum adiponectin levels were measured using commercial enzyme-linked immunosorbent assays (B-Bridge International Inc., Cupertino, CA, USA; Otsuka Pharmaceutical Co., Ltd., Tokyo, Japan). The optical density of each sample was measured using a Bio Rad model 680 spectrophotometer (Bio-Rad Laboratories, Hercules, CA, USA).

The mRNA expression levels of leptin, adiponectin, and PPARγ in epididymal adipose tissue were analyzed by real-time polymerase chain reaction (RT-PCR). The expression levels of Adipo-R1, Adipo-R2, and cadherin 13 in skeletal muscle were also determined by RT-PCR. Briefly, total RNA was isolated using an RNeasy Mini Kit (Qiagen) and cDNA was synthesized using an ExScript RT reagent kit (Takara Bio). RT-PCR was performed using the SYBR Premix Ex Taq (Takara Bio) on an ABI 9700HT (Applied Biosystems) with PCR primers purchased from Takara Bio.

### Statistical analysis

Statistical analysis was carried out using Student’s *t*-test for blood pressure and biochemical factors. Wilcoxon’s test was used for the incidence of stroke.

## Abbreviations

Adipo-R: Adiponectin receptor; ARB: Angiotensin II type I receptor blocker; SHR: Spontaneously hypertensive rat; SHRSP: Stroke-prone spontaneously hypertensive rat; PPARγ: Peroxisome proliferator-activated receptor γ; PBS: Phosphate-buffered saline; RT-PCR: Real-time polymerase chain reaction; RAS: Renin angiotensin system; WKY: Wistar–Kyoto rat.

## Competing interests

The authors declare that they have no competing interests.

## Authors’ contributions

Design of the study: HI; data collection and analysis: KT and TI; final approval of the manuscript: KT, TI, and HI.

## References

[B1] Sequra J Ruilope LM Obesity, essential hypertension and renin-angiotensin system Public Health Nutr 2007 10 1151 1155 17903324 10.1017/S136898000700064X

[B2] Sattler FR Qian D Louie S Johmson D Briggs W DeQuattro V Dube MP Elevated blood pressure in subjects with lipodystrophy AIDS 2001 15 2001 2010 10.1097/00002030-200110190-00013 11600829

[B3] Crane HM Grunfeld C Harrington RD Kitahata MM Lipoatrophy and lipohypertrophy are independently associated with hypertension HIV Med 2009 10 496 503 10.1111/j.1468-1293.2009.00720.x 19486188 PMC2729358

[B4] Auclair M Vigouroux C Boccara F Capel E Vigeral C Guerci B Lascols O Capeau J Caron-Debarle M Peroxisome proliferator-activated receptor-γ mutations responsible for lipodystrophy with severe hypertension activate the cellular renin-angiotensin system Arterioscler Thromb Vasc Biol 2013 33 829 838 10.1161/ATVBAHA.112.300962 23393388

[B5] Lamounier-Zepter V Bornstein SR Kunes J Zicha J Kresk M Ehrhanrt-Bornstein M Ziegler CG Kiessling A Funk RH Haluzik M Adrenocortical changes and arterial hypertension in lipoatrophic A-ZIP/F-1 mice Mol Cell Endocrinol 2008 280 39 46 10.1016/j.mce.2007.09.012 18045774

[B6] Takemori K Gao YJ Ding L Lu C Su LY An WS Vinson C Lee RM Elevated blood pressure in transgenic lipoatrophic mice and altered vascular function Hypertension 2007 49 365 372 17200435 10.1161/01.HYP.0000255576.16089.b9

[B7] Engeli S Schling P Gorzelniak K Boschmann M Janke J Ailhaud G Teboul M Massiera F Sharma AM The adipose-tissue renin-angiotensin-aldosterone system: role in the metabolic syndrome? Int J Biochem Cell Biol 2003 35 807 825 10.1016/S1357-2725(02)00311-4 12676168

[B8] Zorad S Dou JT Benicky J Hutanu D Tybitanclova K Zhou J Saavedra JM Long-term angiotensin II AT1 receptor inhibition produces adipose tissue hypotrophy accompanied by increased expression of adiponectin and PPARgamma Eur J Pharmacol 2006 552 112 122 10.1016/j.ejphar.2006.08.062 17064684 PMC1764497

[B9] Takemori K Ishida H Ito H Continuous inhibition of the renin-angiotensin system and protection from hypertensive end-organ damage by brief treatment with angiotensin II type 1 receptor blocker in stroke-prone spontaneously hypertensive rats Life Sci 2005 77 2233 2245 10.1016/j.lfs.2004.12.048 15963533

[B10] Hamaguchi R Takemori K Inoue T Masuno K Ito H Short-term treatment of stroke-prone spontaneously hypertensive rats with an AT1 receptor blocker protects against hypertensive end-organ damage by prolonged inhibition of the renin-angiotensin system Clin Exper Pharmacol Physiol 2008 35 1151 1155 10.1111/j.1440-1681.2008.04973.x 18518883

[B11] Nishimura Y Ito T Saavedra JM Angiotensin II type 1 blockade normalizes cerebrovascular autoregulation and reduces cerebral ischemia in spontaneously hypertensive rats Stroke 2000 31 2478 2486 10.1161/01.STR.31.10.2478 11022082

[B12] Ito T Nishimura Y Saavedra JM Pre-treatment with candesartan protects from cerebral ischemia J Renin Angiotensin Aldosterone Syst 2001 2 174 179 10.3317/jraas.2001.024 11881119

[B13] Yamakawa H Jezzova M Ando H Saavedra JM Normalization of endothelial and inducible nitric oxide synthase expression in brain microvessels of spontaneously hypertensive rats by angiotensin II AT1 receptor inhibition J Cereb Blood Flow Metab 2003 23 371 380 12621312 10.1097/01.WCB.0000047369.05600.03

[B14] Janke J Engeli S Gorzelniak K Luft FC Sharma AM Mature adipocytes inhibit in vitro differentiation of human preadipocytes via angiotensin type 1 receptors Diabetes 2002 51 1699 1707 10.2337/diabetes.51.6.1699 12031955

[B15] Massiera F Bloch-Faure M Ceiler D Murakami K Fukamizu A Gasc JM Quignard-Boulange A Negrel R Ailhaud G Seydoux J Meneton P Teboul M Adipose angiotensinogen is involved in adipose tissue growth and blood pressure regulation FASEB J 2001 15 2727 2729 11606482 10.1096/fj.01-0457fje

[B16] Cabassi A Coghi P Govoni P Barouhiel E Speroni E Cavazzini S Cantoni AM Scandroglio R Fiaccadori E Sympathetic modulation by carvedilol and losartan reduces angiotensin II-mediated lipolysis in subcutaneous and visceral fat J Clin Endocrinol Metab 2005 90 2888 2897 10.1210/jc.2004-1995 15741261

[B17] Yvan-Charvet L Even P Bloch-Faure M Guerre-Millo M Moustaid-Moussa N Ferre P Quignard-Boulange A Deletion of the angiotensin type 2 receptor (AT2R) reduces adipose cell size and protects from diet-induced obesity and insulin resistance Diabetes 2005 54 991 999 10.2337/diabetes.54.4.991 15793237

[B18] Shimabukuro M Koyama K Chen G Wang MY Trieu F Lee Y Newgard CB Unger RH Direct antidiabetic effect of leptin through triglyceride depletion of tissues Proc Natl Acad Sci USA 1997 94 4637 4641 10.1073/pnas.94.9.4637 9114043 PMC20776

[B19] Avraham Y Davidi N Porat M Chernoguz D Magen I Vorobeiv L Berry EM Leker RR Leptin reduces infarct size in association with enhanced expression of CB2, TRPV1, SIRT-1 and leptin receptor Current Neurovasc Res 2010 7 136 143 10.2174/156720210791184943 20374198

[B20] Oral EA Simha V Ruiz E Andewelt A Premkumar A Snell P Wagner AJ DePaoli AM Reitman ML Taylor SI Gorden P Garg A Leptin-replacement therapy for lipodystrophy N Engl J Med 2002 346 570 578 10.1056/NEJMoa012437 11856796

[B21] Ebihara K Masuzaki H Nakao K Long-term leptin-replacement therapy for lipoatrophic diabetes N Engl J Med 2004 351 615 616 10.1056/NEJM200408053510623 15295061

[B22] Brennan AM Mantzoros CS Drug insight: the role of leptin in human physiology and pathophysiology–emerging clinical applications Nat Clin Pract Endocrinol Metab 2006 2 318 327 10.1038/ncpendmet0196 16932309

[B23] Ebihara K Ogawa Y Masuzaki H Shintani M Miyanaga F Aizawa-Abe M Hayashi T Hosoda K Inoue G Yoshimasa Y Gavrilova O Reitman ML Nakao K Transgenic overexpression of leptin rescues insulin resistance and diabetes in a mouse model of lipoatrophic diabetes Diabetes 2001 50 1440 1448 10.2337/diabetes.50.6.1440 11375346

[B24] Trayhurn P Beattie JH Physiological role of adipose tissue: white adipose tissue as an endocrine and secretory organ Proc Nutr Soc 2001 60 329 339 10.1079/PNS200194 11681807

[B25] Sharma AM Janke J Gorzelniak K Engeli S Luft FC Angiotensin blockade prevents type 2 diabetes by formation of fat cells Hypertension 2002 40 609 611 10.1161/01.HYP.0000036448.44066.53 12411451

[B26] Ardiansyah H Shirakawa H Koseki T Hiwatashi K Takahasi S Akiyama Y Komai M Novel effect of adenosine 5’-monophosphatase on ameliorating hypertension and the metabolism of lipoids and glucose in stroke-prone spontaneously hypertensive rats J Agri Food Chem 2011 59 13238 45 10.1021/jf203237c 22103713

[B27] DeClercq V Taylor CG Wigle J Wright B Tworek L Zahradka P Conjugated linoleic acid improve blood pressure by increasing adiponectin and endothelial nitric oxide synthase activity J Nutr Biochem 2012 23 487 93 10.1016/j.jnutbio.2011.02.003 21684141

